# STARS Is Essential to Maintain Cardiac Development and Function *In Vivo* via a SRF Pathway

**DOI:** 10.1371/journal.pone.0040966

**Published:** 2012-07-18

**Authors:** Nelson W. Chong, Andrea L. Koekemoer, Samir Ounzain, Nilesh J. Samani, Jordan T. Shin, Stanley Y. Shaw

**Affiliations:** 1 Department of Cardiovascular Sciences, Glenfield Hospital, Clinical Sciences Wing, University of Leicester, Leicester, United Kingdom; 2 Cardiology Division, Cardiovascular Research Center, Massachusetts General Hospital and Harvard Medical School, Charlestown, Massachusetts, United States of America; 3 Center for Systems Biology, Simches Research Center, Massachusetts General Hospital, Boston, Massachusetts, United States of America; 4 Department of Medicine, Harvard Medical School, Boston, Massachusetts, United States of America; Virginia Commonwealth University Medical Center, United States of America

## Abstract

**Background:**

STARS (STriated muscle Activator of Rho Signaling) is a sarcomeric protein expressed early in cardiac development that acts as an acute stress sensor for pathological remodeling. However the role of STARS in cardiac development and function is incompletely understood. Here, we investigated the role of *STARS* in heart development and function in the zebrafish model and *in vitro*.

**Methodology and Principal Findings:**

Expression of zebrafish *STARS* (*zSTARS*) first occurs in the somites by the 16 somite stage [17 hours post fertilization (hpf)]. *zSTARS* is expressed in both chambers of the heart by 48 hpf, and also in the developing brain, jaw structures and pectoral fins. Morpholino-induced knockdown of *zSTARS* alters atrial and ventricular dimensions and decreases ventricular fractional shortening (measured by high-speed video microscopy), with pericardial edema and decreased or absent circulation [abnormal cardiac phenotypes in 126/164 (77%) of morpholino-injected embryos vs. 0/152 (0%) of control morpholino embryos]. Co-injection of *zsrf* (serum response factor) mRNA rescues the cardiac phenotype of *zSTARS* knockdown, resulting in improved fractional shortening and ventricular end-diastolic dimensions. Ectopic over-expression of STARS in vitro activates the STARS proximal promoter, which contains a conserved SRF site. Chromatin immunoprecipitation demonstrates that SRF binds to this site *in vivo* and the SRF inhibitor CCG-1423 completely blocks STARS proximal reporter activity in H9c2 cells.

**Conclusions/Significance:**

This study demonstrates for the first time that STARS deficiency severely disrupts cardiac development and function *in vivo* and revealed a novel STARS-SRF feed-forward autoregulatory loop that could play an essential role in *STARS* regulation and cardiac function.

## Introduction

Pathological cardiac remodeling due to sustained mechanical stress, tissue injury and neurohormonal stimulus can lead to hypertrophic growth, cardiomyopathy and heart failure. In order to decipher the complexity of the multiple gene networks that converge to initiate and coordinate such response, essential upstream integral factors need to be identified and characterized. STARS (STriated muscle Activator of Rho Signaling [also known as MS1 (Myocyte Stress 1) and ABRA (Actin-Binding Rho Activating-protein)], is an evolutionarily-conserved sarcomeric actin binding protein that is acutely (and transiently) up-regulated in response to pressure overload left ventricular hypertrophy with a peak expression well before any detectable increase in left ventricular (LV) mass [1], [2]. This suggests a possible role for STARS in the initial signaling of cardiac remodeling such as the hypertrophic response. Sustained over-expression of STARS in the mouse heart have no effect on LV mass but resulted in an increased sensitivity to hemodynamic stress leading to cardiac hypertrophy and heart failure [3].

STARS expression parallels the development and regression of skeletal muscle hypertrophy in humans [4]. Moreover, STARS can stimulate serum response factor (SRF)-dependent transcription *in* cultured cells by inducing the nuclear accumulation of the SRF co-factors, myocardin-related transcription factors (MRTFs) through a mechanism dependent on RhoA (ras homolog gene family, member A) and actin polymerization [2], [5]. Forced over-expression of STARS *in vitro* results in an increase in cell size, induces expression of several MRTF/SRF target genes and provides protection against apoptosis [6], providing further evidence of its role in striated muscle pathophysiology.

*STARS* is expressed during embryonic cardiac development [1], [2] and has been implicated as a key factor involved in myogenic differentiation [7]. STARS could play a more widespread role in muscle pathophysiology such as fluid shear stress induced blood vessel formation (arteriogenesis) [8], insulin resistance and fat metabolism in skeletal muscle [9], [10]. To gain a better understanding on the function and mechanism of STARS in cardiac biology *in vivo*, we investigated the orthologue in zebrafish and report that knockdown of STARS in zebrafish results in severe contractile dysfunction that can be rescued by SRF. We also provide evidence indicating that SRF can regulate *STARS* transcription establishing a newly discovered autoregulatory feed-forward loop for STARS-SRF signaling.

## Results

### Expression of *zSTARS* in the Zebrafish

**Figure 1:**
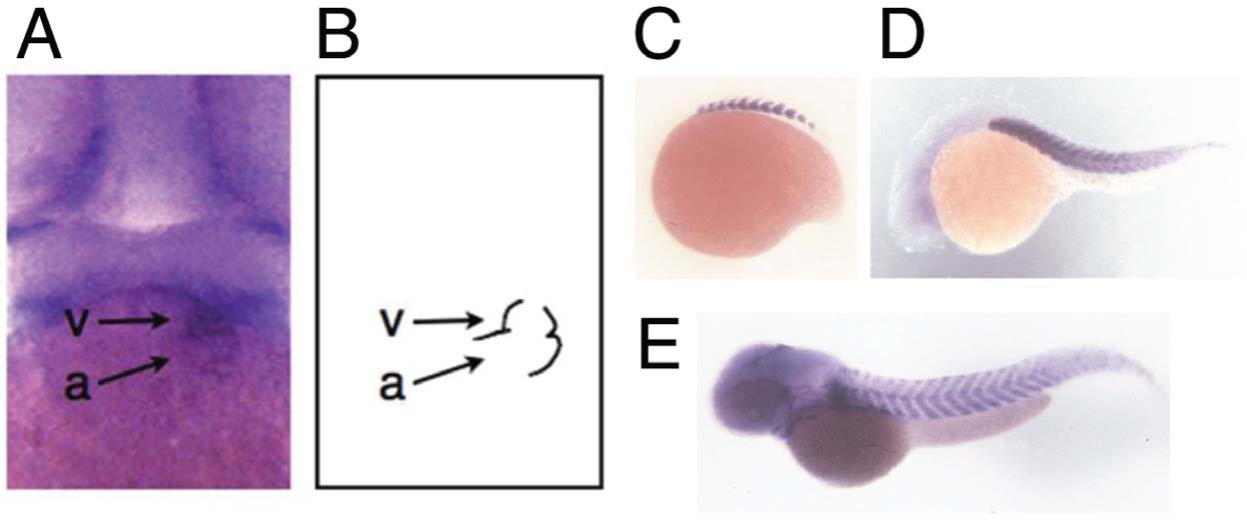
Whole mount *in situ* expression of *zSTARS* in zebrafish embryos. A. Frontal view of 48 hpf embryo showing *zSTARS* expression in ventricle (v) and atrium (a). B. Schematic depicting position of ventricular (v) and atrium (a) in the view from part A. C. Expression in somites at 16 somite stage (17 hpf). D. and E. Expression in somites and central nervous system at 24 hpf (D) and 48 hpf (E).

The zebrafish *STARS* orthologue (zSTARS) contains a high degree of homology to other *STARS* orthologues, particularly in the actin-binding regions critical for signaling from actin to SRF-mediated gene transcription (Figure S1, Text S1). Whole mount *in situ* hybridization was performed in order to investigate the developmental expression pattern of *zSTARS*. *zSTARS* expression was detected in both the atrium and ventricle of the zebrafish heart beginning at 48 hpf (Figure 1A–B). *zSTARS* transcript was also detected in somites by the 16 somite stage (17 hpf) (Figure 1C–E). Other notable embryonic sites of expression include the developing brain (Figure 1D, E), jaw structures, and pectoral fins (data not shown).

### Knockdown of zSTARS in Zebrafish Leads to Structural and Functional Cardiac Abnormalities

**Figure 2:**
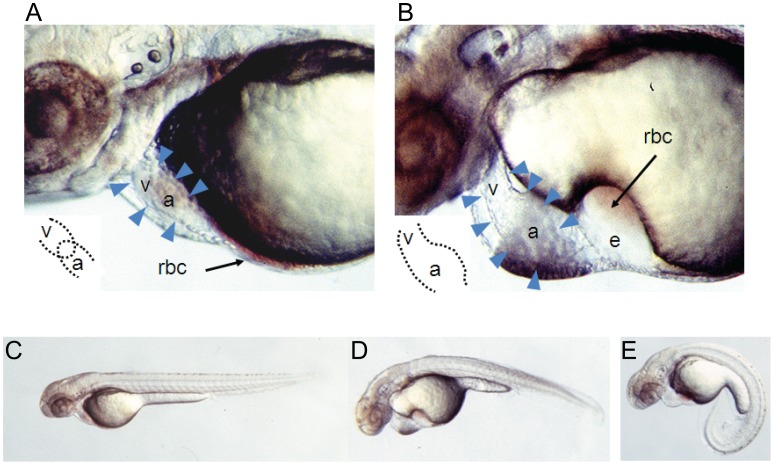
Developmental phenotype of morpholino-induced knockdown of zSTARS: lateral views of 56 hpf embryos. A. Embryo injected with control morpholino (with 5 mismatches). The cardiac silhouette is demarcated by arrowheads. The heart tube is looped so that the ventricle (v) and atrium (a) are closely apposed (inset). Circulating red blood cells (rbc) are visible in a thin rim along the inferior aspect of the yolk and within the heart. B. Embryo injected with *zSTARS* morpholino. The heart tube is unlooped so that the ventricle (v) and atrium (a) are co-linear, with atrial dilation (inset). There is significant edema in the pericardium and over the yolk, with stasis of red blood cells (rbc) over the yolk. C. Lateral view of entire 56 hpf embryo following injection of control, mismatch morpholino. D. and E. 56 hpf embryos showing representative phenotypes of *zSTARS* morpholino injection.

To assess the *in vivo* role of zSTARS during cardiac development, we performed morpholino-induced knockdown of *zSTARS* during zebrafish embryonic development. Injection of a morpholino directed against the translation initiation site of *zSTARS* causes a striking cardiovascular phenotype by 48–56 hpf (126 out of 164 embryos, 77%). Morphologically, the heart tube is incompletely looped, causing the atrium and ventricle to retain an immature linear form (Figure 2A and B, Figure S2A and S2B). The atrium is dilated, and overall contractility of both chambers appears impaired (Movie S1, Movie S2). These cardiac abnormalities lead to significant pericardial edema, with congestion and pooling of blood in the sinus venosus. The most significantly affected embryos show complete absence of circulation despite a beating heart. *zSTARS* morphants also exhibited abnormal somite formation and curvature of the longitudinal axis, suggesting an important developmental role for zSTARS in the non-cardiac sites of expression (Figure 2C–E). In contrast, injection of a control morpholino with five mismatches to the zSTARS sequence does not cause obvious developmental abnormalities (0 out of 152 embryos; χ^2^ = 194, p<0.001) or obstruction of the outflow tract (Figure S2C).

### Over-expression of SRF Rescues the Cardiac Phenotype Induced by *zSTARS* Knockdown

**Figure 3:**
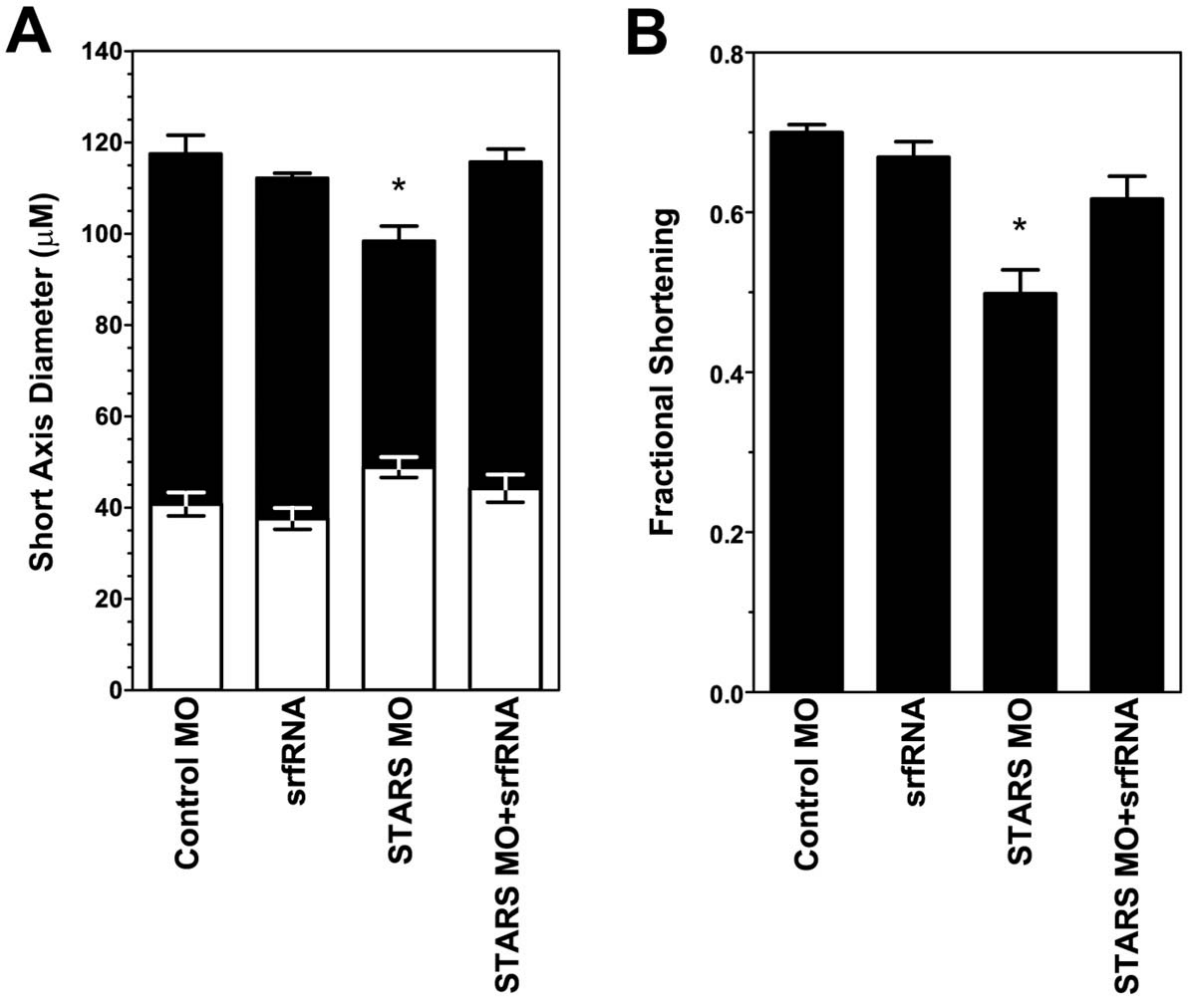
Ventricular function and dimensions based on quantitative analysis of high-speed video microscopy. A. Ventricular dimensions at end-diastole (black bars) and end-systole (white bars). Conditions are identical to those in part a. Values plotted are mean (n = 4 embryos) ± standard deviation. Asterisk (*) denotes statistically significant difference by ANOVA. B. Ventricular fractional shortening observed after injection of: control mismatched morpholino (MM MO), *zSTARS* morpholino + *srf* mRNA (MO + SRF), *srf* mRNA only (SRF only), or *zSTARS* morpholino only (MO only). Values plotted are mean (n = 4 embryos) ± standard deviation. Asterisk (*) denotes statistically significant difference by ANOVA.

We quantified cardiac function and dimensions in *zSTARS* morphants using high-speed video microscopy [11]. In addition to the abnormal morphology of heart development, zSTARS knockdown results in a smaller ventricle. Specifically, ventricular end diastolic size is decreased compared to controls. At end systole, the morphant ventricle is unable to contract to the same degree as control ventricles resulting in a larger ventricular end diastolic size (Figure 3A, Figure S3, Movie S1, Movie S2). Together, these changes result in impaired cardiac performance shown by a significant decrease in ventricular fractional shortening compared to control (Figure 3B). Injection of *zsrf* mRNA suppresses the cardiac phenotype induced by zSTARS knockdown: fractional shortening (p<0.05) and ventricular systolic and diastolic dimensions are restored to values indistinguishable from control embryos (Figure 3, Movie S3).

### SRF Regulate STARS Transcription *via* an Autoregulatory Feed-forward Loop

**Figure 4:**
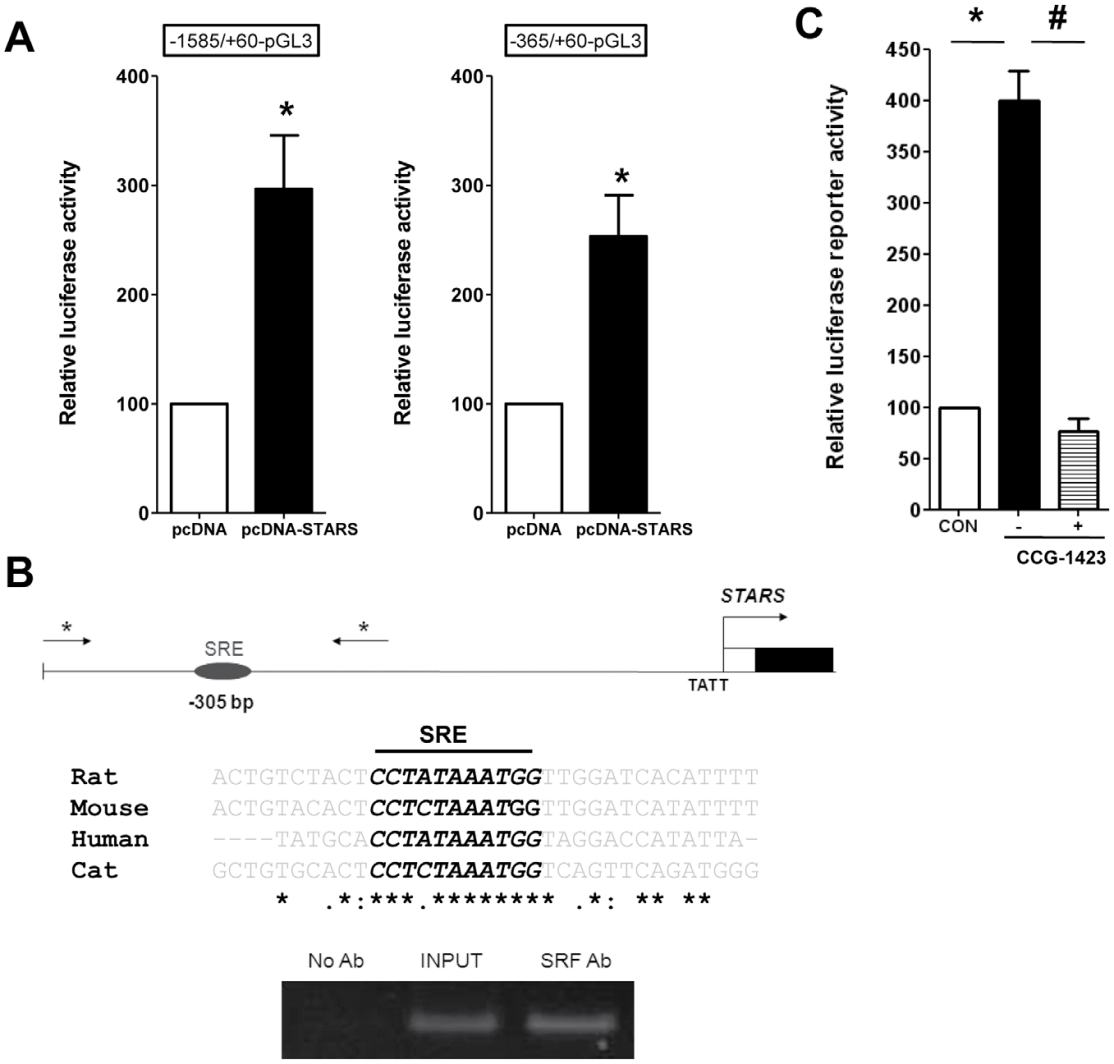
Binding of SRF to the *STARS* promoter. A. STARS expression activates reporter gene constructs containing the conserved serum response element (SRE). Luciferase activity is shown for two constructs upstream of the *STARS* transcription start site. B. Chromatin immunoprecipitation assays were performed with formaldehyde cross-linked chromatin isolated from feline adult cardiomyocytes. Asterisk (*) denotes PCR primer locations. Immunoprecipitations were performed without primary antibody (No Ab) as a negative control, with anti-SRF antibody. Input DNA is also shown as a positive control. Similar results were observed in four independent experiments. C. The SRF inhibitor CCG-1423 (1 µM) abolished *STARS* −365/+60 promoter-reporter activity in H9c2 cells (n = 3 experiments, in triplicates).

Ectopic over-expression of STARS significantly increased the activity of the *STARS* proximal reporter, which contains a conserved SRF binding site (position −305 bp). *In vivo* binding of SRF to this site was confirmed by ChIP (Figure 4). This and similar SRF-chromatin preparations have been used to detect SRF binding to the sodium-calcium exchanger (NCX1) gene [12]. The SRF inhibitor CCG-1423, which works by inhibiting MRTF nuclear localization [9], completely blocked *STARS* proximal reporter activity in H9c2 cells. Of interest, over-expression of rat STARS also significantly increased *srf* mRNA and several MRTF-SRF target genes in H9c2 cells [6], [13].

## Discussion

In this study, we isolated the mammalian homologue of *STARS* in zebrafish and examined its role in cardiac function and development. Morpholino knockdown of STARS in the zebrafish results in abnormal cardiac looping, an enlarged atrium, and severe contractile dysfunction with resulting marked impaired circulation.These phenotypes confirm an important role for STARS in cardiac development and function. Notably, the cardiac phenotype of *zSTARS* knockdown resembles that of a zebrafish morpholino against cardiac troponin T (*tnnt2*), which can be mutated in human hypertrophic cardiomyopathy [14]. Both *zSTARS* and *zTnnt2* knockdown result in enlarged atria, decreased ventricular fractional shortening, and decreased end diastolic dimension. Thus, genes such as *STARS* and *Tnnt2* which regulate hypertrophic response in adults may also play critical roles in cardiac embryonic development. Furthermore, these examples illustrate how studying developmental phenotypes arising from zebrafish knockdown can shed light on physiological mechanisms of cardiac stress response (such as cardiomyopathy and contractility dysfunction).

Previous studies have demonstrated that STARS can activate *srf* transcription activity *in vitro* [2], [3]. In the present study, we provide the first *in vivo* evidence that *srf* is an essential downstream target of STARS by showing that injection of z*srf* mRNA rescues cardiac abnormalities caused by morpholino-mediated *zSTARS* knockdown. SRF is known to be involved in cardiac development, structure and function [15], [16] and knockdown of *STARS* was shown to reduce SRF activity [5]. In addition, it was demonstrated that cardiac-specific deletion of SRF in the embryonic heart results in cardiac defects [15] and deletion of SRF from the adult heart caused reduced contractility leading to dilated cardiomyopathy [17]. It is conceivable that knockdown of *STARS* decreased SRF activity, which resulted in decreased cardiac function. Consistent with this hypothesis, SRF expression restored SRF activity and rescued the cardiac abnormalities as shown here.

In addition to its effects on cardiac function, *zSTARS* knockdown causes phenotypes consistent with its non-cardiac sites of expression, including abnormal somite formation and curvature and shortening of the longitudinal axis. As the axial skeleton and skeletal muscle are derived from somites [18], this novel finding implies an important role for STARS in skeletal muscle development. STARS levels were up-regulated in human skeletal hypertrophy and down-regulated in skeletal muscle atrophy [4] and in aged skeletal muscle of mice [19] demonstrating a role for STARS in the maintenance of skeletal muscle. Interestingly, the same finding was observed for SRF [4], [19] and deletion of SRF in mice resulted in impaired muscle growth [20]. Collectively, these results extend the findings in cardiac tissue and suggest that the STARS-SRF signalling pathways are similarly involved in skeletal muscle development and function.

The present study demonstrates for the first time that *STARS*-deficient animals display severe abnormalities in cardiac development and function, which were rescued by SRF over-expression. Our study further highlights the importance of sarcomeric proteins in cardiac development and biology [21], [22]. Both STARS and SRF play comparable roles in not just establishing and maintaining cardiac physiology but also in skeletal muscle and brain development and function. These findings provide support for a widespread role of the STARS-SRF signaling axis in multiple tissues *in vivo*. Understanding the precise mechanism of STARS expression and regulation could provide a novel avenue to dissect the initiation process of cardiac dysfunction and heart failure.

## Materials and Methods

### Zebrafish Experiments

All zebrafish experiments were approved by the Subcommittee on Research Animal Care at Massachusetts General Hospital. The investigation conformed to the *Guide for the Care and Use of Laboratory Animals* published by the US National Institutes of Health (NIH Publication No. 85-23, revised 1996). IACUC approval number A3596-01. Zebrafish (AB strain) were raised and maintained using standard protocols.

An antisense morpholino oligonucleotide was designed against the translation initiation site, with the sequence: GCTGTACTCATGGTGTTTTAATTTG (Gene-Tools, Philomath, OR). Antisense and scrambled morpholinos were injected according to standard procedures [23]. For expression of zebrafish (z) SRF, the T7 phage promoter sequence was added to the 5′ end of the *zsrf* cDNA (Open Biosystems, Huntsville, AL) by PCR; *zsrf* mRNA was synthesized by *in vitro* transcription and injected using published methods [11], [23], [24].

### Cloning of Zebrafish *STARS* Orthologue (*zSTARS*)

Comparison of the rat *STARS* sequence with zebrafish sequences identified a homologous EST (AI721847). Primers were designed within this sequence for nested 5′ and 3′ RACE PCR reactions (SMART RACE, Clontech) on cDNA isolated from 72 hpf (hours post fertilization) zebrafish embryos (Trizol (Invitrogen), according to manufacturer’s instructions). 3′ RACE PCR primer sequences were as follows: 5′-CGCCGAAGTGTAACGAGTTTGGAAAG-3′ and 5′-CACCAATCAACTGACGACTGAAGACACC-3′. 5′ RACE PCR primer sequences were as follows: 5′-GAGCTTCTGGCCCTCCATGTGATCT-3′ and 5′-ATCGCTGTTGTCGCTCAGAGATGCT-3′. Full length coding sequence of *zSTARS* was cloned into pCRIITopo using the TopoTA cloning system (Invitrogen).

### *In situ* Hybridization

Digoxigenin labeled RNA probes were generated by *in vitro* transcription according to manufacturer’s instructions (MEGAscript SP6, Ambion). Embryos were fixed at the indicated times in 4% formaldehyde in PBS overnight at 4°C, and transferred to methanol for storage at −20°C. *In situ* hybridization on whole mount embryos was performed as previously described [11].

### Antisense Morpholino Injection

An antisense morpholino oligonucleotide was designed against the translation initiation site, with the sequence: GCTGTACTCATGGTGTTTTAATTTG (Gene-Tools). A control morpholino with 5 mismatches was also designed: GCTcTACTgATGcTGTTTaAATaTG. Morpholinos were dissolved in Danieau’s buffer according to manufacturer’s instructions at a concentration of 300 µM, and injected into embryos at the 1 cell stage [23].

### RNA Injection

The T7 phage promoter sequence was added to the 5′ end of the cDNA encoding *zsrf* (Open Biosystems, Huntsville, AL) by PCR. This template was used to synthesize capped mRNA encoding the full-length coding sequence of *srf* by *in vitro* transcription (mMessage Machine, Ambion). mRNA was diluted in Danieau’s buffer to a final concentration of 100 ng/µL and co-injected with morpholino (at 400 µM) as above. n = 4 for each condition.

### Ventricular Function Measurements and M-mode Recordings

Extensive description on the measurement of cardiovascular function and the creation of m-mode images have been published elsewhere. Ventricular size and fractional shortening were measured as previously described [24].

### STARS Promoter-reporter Transfection Assays and mRNA Analysis

The rat *STARS*−1585/+60-Luc reporter was generated as described elsewhere [7]. For the −365/+60 region, the rat *STARS* 5-upstream region was PCR-amplified using primers -365-MluI-Forward: 5′-GTACGCGTTACAGAGGTTTAAGTGAGAGC -3′ and +60-BglII-Reverse: 5′- CCAGATCTCAGGCTACCTGTTTCTTCTC-3′, gel-purified and cloned into pGEMTEasy vector (Promega). This plasmid was subsequently restriction digested by *Mlu*I and *BglII* and the insert was gel-purified and subcloned directionally into the pGL3-Promoter Luc vector (Promega). The *STARS*-365/+60 Luc reporter was sequenced for authenticity.

Transfection of *STARS* Luciferase reporters were done as described [7]. Briefly, 0.5 ug of *STARS* reporter plasmids (or its empty control vector pGL3-promoter) and 10 ng of Renilla reporter was cotransfected into H9c2 cells (60% confluency) in 24-well plates using the JetPei reagent (Source BioScience). For standard transfection, cell extracts were measured for activity after 48 h using a Dual-Luciferase Assay kit (Promega). For SRF inhibition experiments, CCG-1423 (1 µM; Sigma) was added 24 h post-transfection and left for a further 24 h before assayed.

### Chromatin Immunoprecipitation (ChIP)

The feline cardiomyocyte chromatin was a gift from Dr DL Menick [12], [25] obtained using the Santa Cruz SRF G-20 antibody (sc-335). The feline *STARS* proximal promoter was PCR amplified from the immunoprecipitated and non-immunoprecipitated chromatin (negative control) using the following primers: STARS-PP-Fw Sense: 5′-CGGAGCTCAGAACACCGTCAGAGCCATAGC-3′, and STARS-PP-Rv Antisense- 5′-CCAAGCTTCAGGCTACCTGTTTCTTCTC-3′. The input DNA was used as the positive control.

### Statistical Analysis

Data are presented as mean±SEM. Data were analyzed by ANOVA followed by Bonferroni post hoc testing (Graph Pad Prism, La Jolla, CA).

## Supporting Information

Figure S1 **Amino acid alignment of human, mouse, rat and zebrafish STARS.**
(DOC)

Figure S2 **Tg(FLK:G-RFP) embryos were injected with MO at the 1 cell stage and allowed to develop under standard conditions.**
(DOC)

Figure S3 **M-mode images of zebrafish ventricles.**
(DOC)

Movie S1 **Cardiac ventricular function of control mismatched** ***zSTARS*** **morphants.**
(MOV)

Movie S2 **Ventricular function of** ***STARS*** **morphants.**
(MOV)

Movie S3 **Ventricular function of** ***zSTARS*** **morpholino with** ***zsrf*** **mRNA.**
(MPG)

Text S1 (DOC)
