# Supplementary material for: STARS Is Essential to Maintain Cardiac Development and Function In Vivo via a SRF Pathway
Source: PLoS One. 2012 Jul 18;7(7):e40966. doi: 10.1371/journal.pone.0040966 (PMC3399798; doi:10.1371/journal.pone.0040966)
Supplement: Figure S1 — Amino acid alignment of human, mouse, rat and zebrafish STARS. (DOC) [file pone.0040966.s001.doc]

**Chong et al. Figure S1**

1 50

H. sapiens (1) MAPGEKESGEGPAKSALRKIRTATLVISLARGWQQWANENSIRQAQEPTG

M. Musculus (1) MAPGEREREAGPAKSALRKVRTATLVINLARGWQQWANENSTKQAQEPAG

R. norvegicus (1) MAPGETVREAGPAKSALQKVRRATLVINLARGWQQWANENSTRQAQEPAG

D. rerio (1) MSTAGVQQNR-PFSRAVRKIKVASTVNSLAKSWQSWANKHSDKQDTIPSG

51 100

H. sapiens (51) WLPGGTQDSPQAPKPITPPTSHQKAQSAPKSPPRLPEGHGDGQSSEKAPE

M. Musculus (51) WLPGATHDVPNAPKEAGP------YQHAPKTLSPKPDRDGEGQHSEEATE

R. norvegicus (51) WLPGATQDLPHTPKEPGP------RQHAPKPPSPKPDGDREGRGSEEATE

D. rerio (50) WMPDTIIEDAKE-------------KQKEKNEMKLLVTPRVVSVVGEEAT

101 150

H. sapiens (101) VSHIKKKEVSKTVVSKTYERGGDVSHLSHRYERDAGVLEPGQPENDIDRI

M. Musculus (95) VSHIKRKEVTRTVVSKAYERGGDVNYLSHRYENDGGVSEAIQPENDIDRI

R. norvegicus (95) VSHIKRKEVTRTVVSKAYERGGDVNYLSHRYEHDGGVSEAVQPDNDIDRI

D. rerio (87) DDQIKTGIVTKAITPKCNEFGKDLVSVIKEKINTN------QLTTEDTKN

151 200

H. sapiens (151) LHSHGSPTRRRKCANLVSELTKGWRVMEQEEPTWRSDSVDTEDSGYGGEA

M. Musculus (145) LLSHDSPTRRRKCTNLVSELTKGWKVMEQEEPTWKSDSVDTEDSGYGGDM

R. norvegicus (145) LLSHDSPTRRRKCTNLVSKLTKGWKVMEQEEPKWKSDSIDTEDSGYGGDM

D. rerio (131) FLGNESPTRRRYCGGKAGTFGKAIGRKEGKSMGSRSSSLDADDSGLGEEA

201 250

H. sapiens (201) EERPEQDGVQVAVVRIKRPLPSQVNRFTEKLNCKAQQKYSPVGNLKGRWQ

M. Musculus (195) EERPEQDAAPVAPARIKRPLHSQANRYSEPLNCKAHRKYSQVDNLKGRWQ

R. norvegicus (195) EERPEQDVAQVAAARIKRPLHSQANRYSETLNCKAHRKYSQVDNLKGRWQ

D. rerio (181) SLSDNSDLNENEPKKHVN---------------RHKIKVTTMGDLRSRWQ

251 300

H. sapiens (251) QWADEHIQSQKLNPFSEEFDYELAMSTRLHKGDEGYGRPKEGTKTAERAK

M. Musculus (245) QWADEHVQSQKLNPFSDEFDYDLAMSTRLHKGDEGYGRPKEGSKTAERAK

R. norvegicus (245) QWADEHIQSQKLNPFSDEFDYDLAMSTRLHKGDEGYGRPKEGSKTAERAK

D. rerio (216) RFAEDHMEGQKLNPFSEEFDYDHAMATRLHKGDAGYGRPKEGSKTAQRAD

301 350

H. sapiens (301) RAEEHIYREMMDMCFIICTMARHRRDGKIQVTFGDLFDRYVRISDKVVGI

M. Musculus (295) RAEEHIYREIMELCFVIRTMARHRRDGKIQVTFGELFDRYVRISDKVVGI

R. norvegicus (295) RAEEHIYREIMELCFVIRTMARHRRDGKIQVTFGELFDRYVRISDKVVGI

D. rerio (266) RAQKHIYREMEEMCFIIRDMGQQDKQGQIWVTFGRLFDRYVKISDKVVGI

351 381

H. sapiens (351) LMRARKHGLVDFEGEMLWQGRDDHVVITLLK

M. Musculus (345) LMRARKHGLVHFEGEMLWQGRDDHVVITLVE

R. norvegicus (345) LMRARKHGLVHFEGEMLWQGKDDHVVITLLE

D. rerio (316) LLRCRKHKMVDFEGEMLWKGQDDDVIIPLLV
