# Supplementary figures and images for: STARS Is Essential to Maintain Cardiac Development and Function In Vivo via a SRF Pathway
Source: PLoS One. 2012 Jul 18;7(7):e40966. doi: 10.1371/journal.pone.0040966 (PMC3399798; doi:10.1371/journal.pone.0040966)

**Chong et al. Figure S2**

**
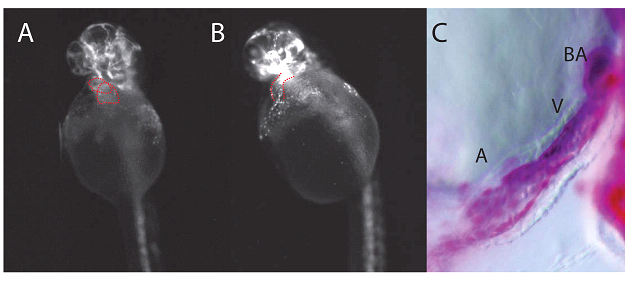
**

Supplement: Figure S2 — Tg(FLK:G-RFP) embryos were injected with MO at the 1 cell stage and allowed to develop under standard conditions. (DOC) [file pone.0040966.s002.doc]

**Chong et al. Figure S3**


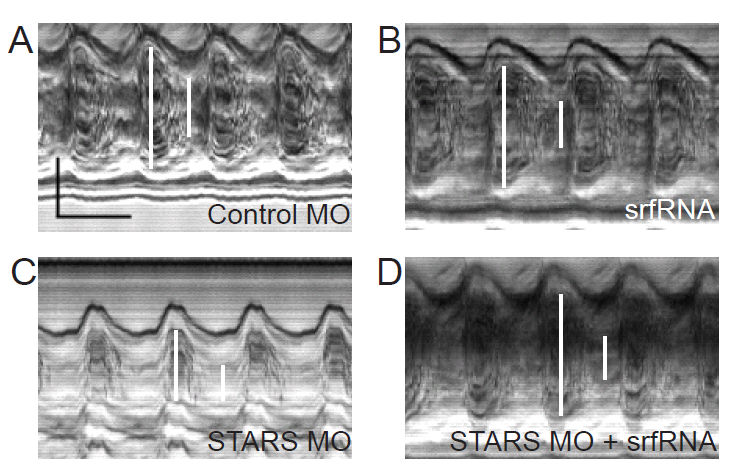

Supplement: Figure S3 — M-mode images of zebrafish ventricles. (DOC) [file pone.0040966.s003.doc]
