# Supplementary material for: STARS Is Essential to Maintain Cardiac Development and Function In Vivo via a SRF Pathway
Source: PLoS One. 2012 Jul 18;7(7):e40966. doi: 10.1371/journal.pone.0040966 (PMC3399798; doi:10.1371/journal.pone.0040966)
Supplement: Text S1 — (DOC) [file pone.0040966.s007.doc]

# Supporting Information

# *STARS is essential to maintain cardiac development and function in vivo via a SRF pathway* (Chong et al.)

**Figure S1. Amino acid alignment of human, mouse, rat and zebrafish STARS.**

The zebrafish *STARS* orthologue (*zSTARS*) encodes a protein of 346 amino acids. There is a high degree of homology to *the H. sapiens, M. musculus*, and *R. norvegicus* orthologs indicating evolutionary conservation. the homology is strongest in the C-terminal portion of the protein, which is the region required STARS to bind actin, as well as to activate SRF-dependent transcription; this C-terminal domain also binds ABLIM-2 and -3, two actin-binding proteins that enhance STARS’s ability to activate SRF-dependent transcription [1]. The regions of STARS critical for signaling from actin to transcriptional activation are conserved in the zebrafish orthologue.

**Figure S2.** Tg(FLK:G-RFP) embryos were injected with MO at the 1 cell stage and allowed to develop under standard conditions [2], and fluorescent and bright-field images of 48 hpf zebrafish are shown. Panel A: Tg(FLK:G-RFP) injected with mismatch MO showing normal cardiac looping. Panel B: Tg(FLK:G-RFP) injected with STARS MO showing defective looping. Panel C: Bright-field image of the heart following digital subtraction of fluorescent Tg(FLK:G-RFP) image: this highlights the cardiac lumen through the atrium (A), ventricle (V) and the bulbus arteriosus (BA), showing that the outflow tract is unobstructed.

**Figure S3. M-mode images of zebrafish ventricles.** Fertilized zebrafish oocytes were injected with (A) control morpholino, (B) *in vitro* transcribed mRNA for *srf* (srfRNA), (C) morpholino targeting *STARS* mRNA (mo*STARS*) or (D) mo*STARS* and *srf*RNA and allowed to develop under standard conditions. High speed video images of ventricles were obtained at 48 hpf and motion mode (m-mode) images obtained at 125 frames per second as described [3]. In these frames, the vertical dimension displays the image across a single scan line over time in the horizontal dimension. Ventricular short axis diameter (white bars) and myocardial thickness were measured at end diastole and end systole in each condition. In panel (A), the vertical black bar denotes 50 microns and the horizontal bar denotes 500 msec.

**References**

1. Barrientos T, Frank D, Kuwahara K, Bezprozvannaya S, Bassel-Duby R, Richardson JA, Katus HA, Olson EN, Frey N. Two novel members of the ABLIM protein family, ABLIM-2 and -3, associate with STARS and directly bind F-actin. *J Biol Chem* 2007;**282**:8393-8403.

# 2. [**Cross LM**](http://www.ncbi.nlm.nih.gov/pubmed?term=Cross LM%5BAuthor%5D&cauthor=true&cauthor_uid=12740225), [**Cook MA**](http://www.ncbi.nlm.nih.gov/pubmed?term=Cook MA%5BAuthor%5D&cauthor=true&cauthor_uid=12740225), [**Lin S**](http://www.ncbi.nlm.nih.gov/pubmed?term=Lin S%5BAuthor%5D&cauthor=true&cauthor_uid=12740225), [**Chen JN**](http://www.ncbi.nlm.nih.gov/pubmed?term=Chen JN%5BAuthor%5D&cauthor=true&cauthor_uid=12740225), [**Rubinstein AL**](http://www.ncbi.nlm.nih.gov/pubmed?term=Rubinstein AL%5BAuthor%5D&cauthor=true&cauthor_uid=12740225). Rapid analysis of angiogenesis drugs in a live fluorescent **zebrafish** assay. [***Arterioscler Thromb Vasc Biol.***](http://www.ncbi.nlm.nih.gov/pubmed/?term=cross l 2003 sebrafish) **2003**;23:911-912.

# 3. [**Shin JT**](http://www.ncbi.nlm.nih.gov/pubmed?term="Shin JT"%5BAuthor%5D), [**Pomerantsev EV**](http://www.ncbi.nlm.nih.gov/pubmed?term="Pomerantsev EV"%5BAuthor%5D), [**Mably JD**](http://www.ncbi.nlm.nih.gov/pubmed?term="Mably JD"%5BAuthor%5D), [**MacRae CA**](http://www.ncbi.nlm.nih.gov/pubmed?term="MacRae CA"%5BAuthor%5D). High-resolution cardiovascular function confirms functional orthology of myocardial contractility pathways in zebrafish. [***Physiol Genomics*.**](http://www.ncbi.nlm.nih.gov/pubmed/20388839) 2010;42:300-309.
